# Supplementary material for: Effects of Methyl Terminal and Carbon Bridging Groups Ratio on Critical Properties of Porous Organosilicate Glass Films
Source: Materials (Basel). 2020 Oct 10;13(20):4484. doi: 10.3390/ma13204484 (PMC7601386; doi:10.3390/ma13204484)
Supplement: Supplementary file 1 [file materials-13-04484-s001.pdf]

materials

Supplementary Materials

Effects of Methyl Terminal and Carbon Bridging Groups Ratio on Critical Properties of Porous Organosilicate-Glass Films

Alexey S. Vishnevskiy [1,*], Sergej Naumov [2], Dmitry S. Seregin [1], Yu-Hsuan Wu [3], Wei-Tsung Chuang [4], Md. Rasadujjaman [5,6], Jing Zhang [5], Jihperng Leu [3], Konstantin A. Vorotilov [1] and Mikhail R. Baklanov [1,5]

1 Research and Education Center "Technological Center", MIREA—Russian Technological University (RTU MIREA), Moscow 119454, Russia; techcenter@mirea.ru
2 The Leibniz Institute of Surface Engineering (IOM), Leipzig 04318, Germany; sergej.naumov@iom-leipzig.de
3 Department of Materials Science and Engineering, National Chiao Tung University, Hsinchu 30049, Taiwan; jimleu@nctu.edu.tw
4 National Synchrotron Radiation Research Center, Hsinchu 30076, Taiwan; weitsung@nsrrc.org.tw
5 Department of Microelectronics, North China University of Technology, Beijing 100144, China; zhangj@ncut.edu.cn
6 Department of Physics, Dhaka University of Engineering & Technology, Gazipur 1700, Bangladesh; rasadphy@duet.ac.bd
* Correspondence: vishnevskiy@mirea.ru

S1. The Data Generated by Spectroscopic Ellipsometer

Table S1. UV-Vis optical characteristic of porous organosilicate glass films with different types and content (25 and 45 mol%) of bridging groups, cured at 430 °C for 30 min in air.

mol %	Type of Bridging Group		
	**Methylene**	**Ethylene**	**1,4-phenylene**
**25**	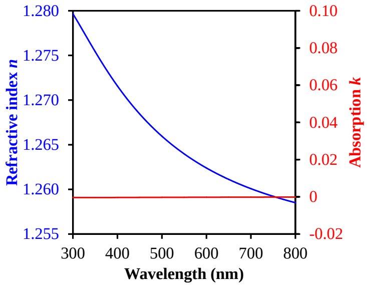	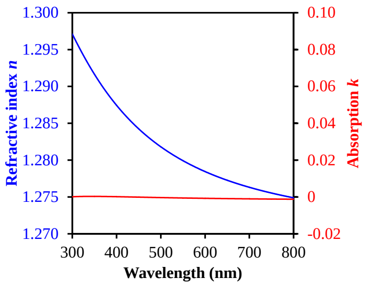	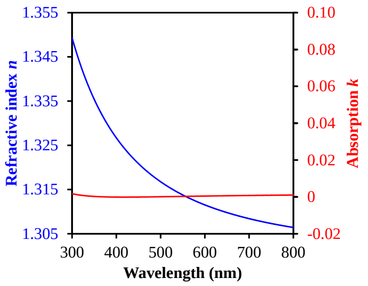
**45**	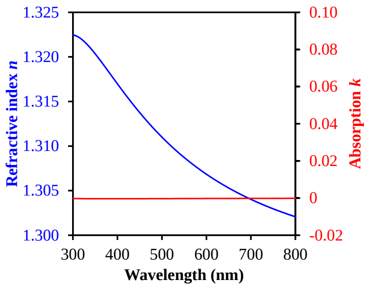	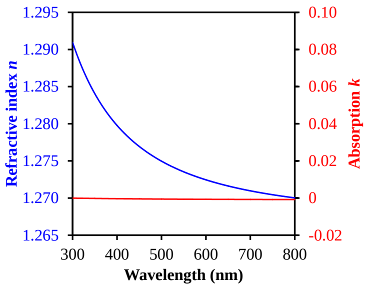	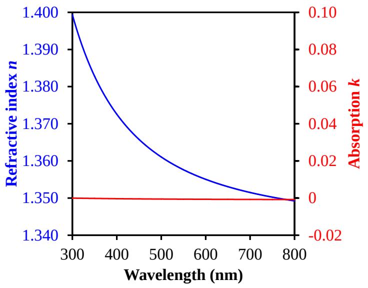

<table>
<tr><th rowspan="2">mol %</th><th colspan="3">Type of Bridging Group</th></tr>
<tr><th>Methylene</th><th>Ethylene</th><th>1,4-phenylene</th></tr>
<tr><td>60</td>
<td>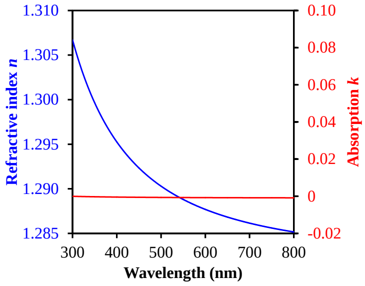
</td>
<td>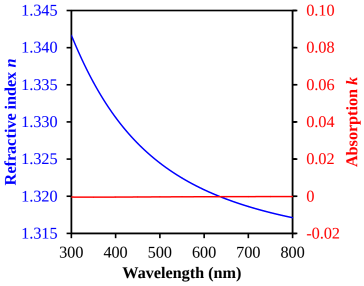
</td>
<td>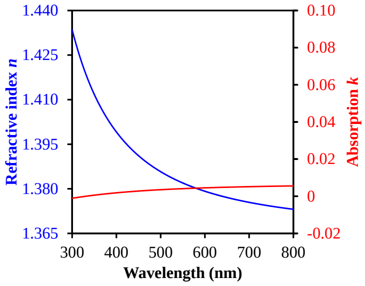
</td></tr>
<tr><td>100</td>
<td>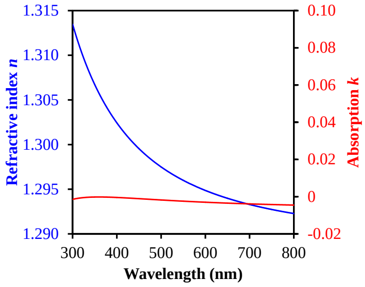
</td>
<td>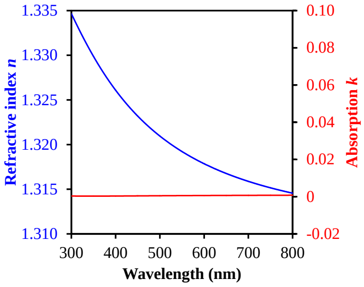
</td>
<td>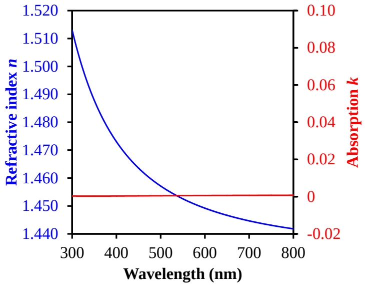
</td></tr>
</table>

The films are transparent in the range of 300–800 nm. UV absorption spectra have been presented in our previous publication (Ref. [24] in the reference list).

S2. The Data Generated by Fourier-Transform Infrared Spectroscopy

Table S2. The summarized FTIR data for porous methylsilsesquioxane film (MSSQ) and organosilicate glass films with different types (M–methylene, E–ethylene, B–1,4-phenylene) and content (0–100 mol%) of bridging groups, annealed at 430 °C for 30 min in air (hard bake). In this Table: MTMS is methyltrimethoxysilane, BTESM is 1,2-bis(triethoxysilyl)methane, BTMSE is 1,2-bis(trimethoxysilyl)ethane, BTESB is 1,4-bis(triethoxysilyl)benzene.

Exp. No	Sample No	Sol Composition (mol%)	$A_{Si–CH_3(\sim1275\ cm^{-1})}/A_{Si–O–Si}$	$A_{Si–OH(\sim950\ cm^{-1})}/A_{Si–O–Si}$
1	MSSQ	MTMS = 100	0.049	0.0006
2	25M	MTMS = 75, BTESM = 25	0.026	0.0009
	25E	MTMS = 75, BTMSE = 25	0.027	0.0030
	25B	MTMS = 75, BTESB = 25	0.026	0.0148
3	45M	MTMS = 55, BTESM = 45	0.021	0.0009
	45E	MTMS = 55, BTMSE = 45	0.018	0.0027
	45B	MTM = 55, BTESB = 45	0.016	0.0251
4	60M	MTMS = 40, BTESM = 60	0.016	0.0029
	60E	MTMS = 40, BTMSE = 60	0.012	0.0078
	60B	MTMS = 40, BTESB = 60	0.012	0.0440
5	100M	MTMS = 0, BTESM = 100	0.013	0.0046
	100E	MTMS = 0, BTMSE = 100	0.005	0.0131
	100B	MTMS = 0, BTESB = 100	0.005	0.0336

With an increase in the bridges' concentration, the expected decrease in the number of methyl groups, and an increase in the amount of non-condensed silanols, are observed.

S3. The Data Generated by Contact Angle Measuring Device

Table S3. Water contact angle values for porous methylsilsesquioxane film (MSSQ) and some of the organosilicate glass films deposited with different ratios of 1,2-bis(trimethoxysilyl)ethane to methyltrimethoxysilane (BTMSE/MTMS) mixture, annealed at 430 °C for 30 min in air (hard bake).

Sample No	BTMSE/MTMS Ratio	Water Contact Angle (deg.)
MSSQ	0/100	102 ± 2
25E	25/75	79 ± 2
45E	45/55	68 ± 2

One can see that MSSQ is the most hydrophobic material; an increase in the content of ethylene bridges decreases the contact angle.

S4. The Data Generated by Ellipsometric Porosimetry

Table S4. Open porosity V_{open} and pore radius (R) distribution measured by ellipsometric porosimetry.

mol %	Type of Bridging Group		
	Methylene	Ethylene	1,4-phenylene
25	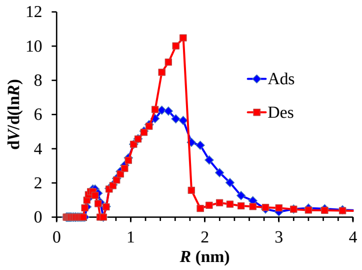 V_{open} = 39.3%<br>R_{ads}/R_{des} = 1.42/1.71 nm	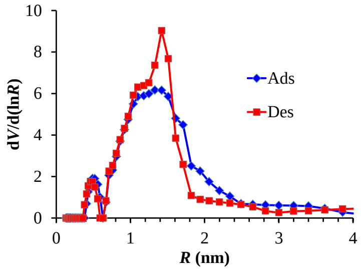 V_{open} = 33.9%<br>R_{ads}/R_{des} = 1.33/1.42 nm	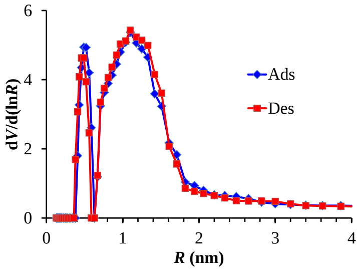 V_{open} = 35.5%<br>R_{ads}/R_{des} = 1.10/1.10 nm
45	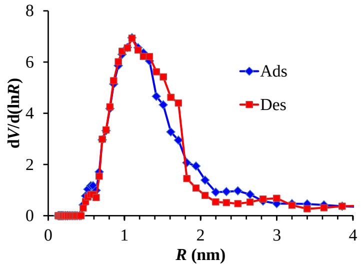 V_{open} = 28.0%<br>R_{ads}/R_{des} = 1.10/1.10 nm	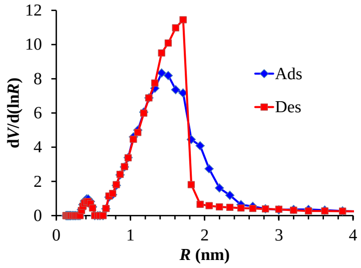 V_{open} = 36.3%<br>R_{ads}/R_{des} = 1.42/1.71 nm	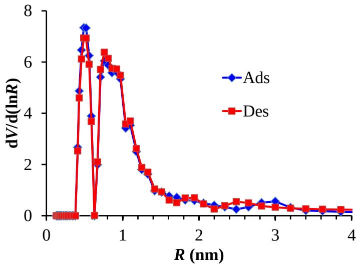 V_{open} = 29.3%<br>R_{ads}/R_{des} = 0.76/0.76 nm
60	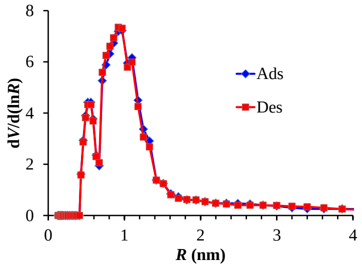 V_{open} = 34.4%<br>R_{ads}/R_{des} = 0.97/0.92 nm	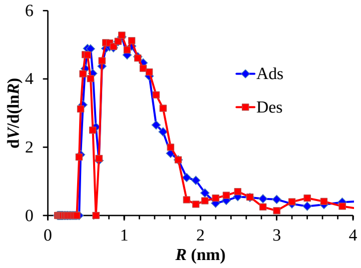 V_{open} = 28.0%<br>R_{ads}/R_{des} = 0.97/0.97 nm	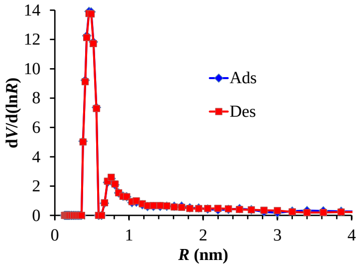 V_{open} = 27.2%<br>R_{ads}/R_{des} = 0.76/0.76 nm

**mol %**	**Type of Bridging Group**		
	**Methylene**	**Ethylene**	**1,4-phenylene**
**100**	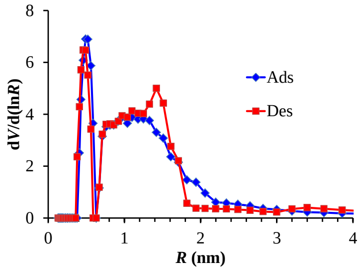<br>V_{open} = 32.8%<br>R_{ads}/R_{des} = 1.10/1.42 nm	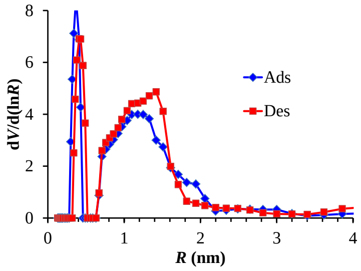<br>V_{open} = 32.1%<br>R_{ads}/R_{des} = 1.18/1.42 nm	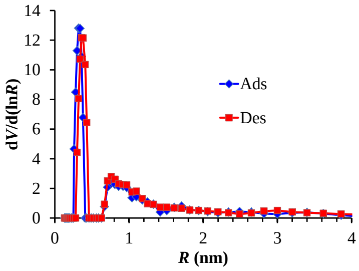<br>V_{open} = 19.6%<br>R_{ads}/R_{des} = 0.76/0.76 nm

In all types of bridging group, one can see a decrease in pore size with an increase in the concentration of the introduced alkylenesiloxane.

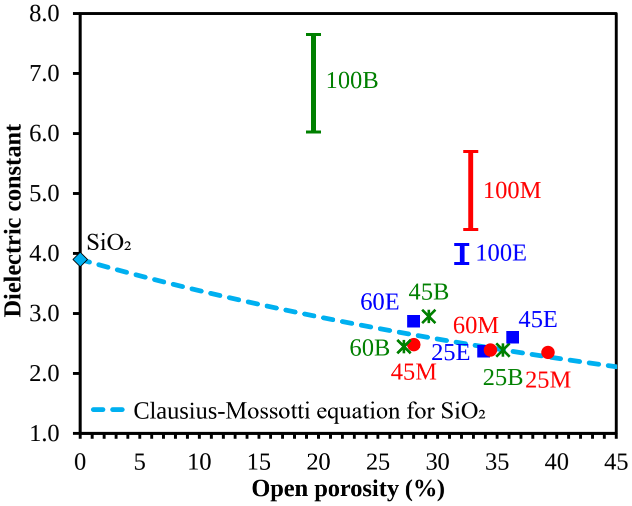

Figure S1. The measured dielectric constants versus porosity and comparison with the Clausius–Mossotti equation plotted for SiO_2.

One can see that hydrophobic samples with 25 mol% bridge concentration are perfectly fitted by this curve while samples 45B, 45E and 60E show slightly higher *k*-values because of the presence of adsorbed water. The samples with 100 mol% bridge concentration without terminal methyl groups are hydrophilic and the measured dielectric constant is much higher than can be expected by the Clausius–Mossotti curve for Silica film. In this Figure: M—methylene bridge (BTESM), E—ethylene bridge (BTMSE), B—1,4-phenylene bridge (BTESB) and the preceding digits indicate the alkylenesiloxane content in the matrix precursor in mol%.

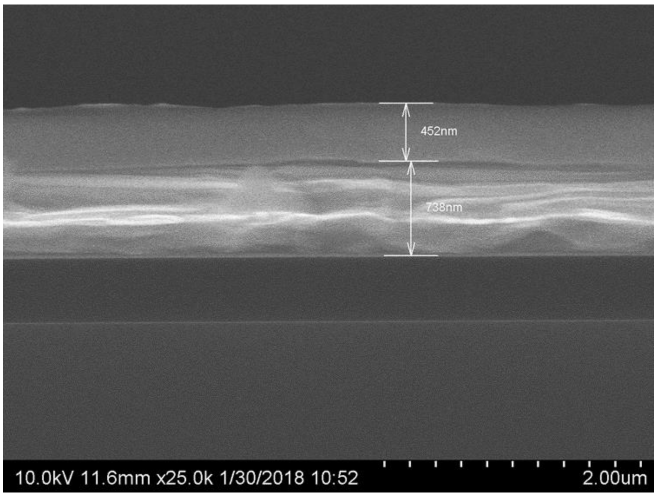

Figure S2. SEM picture of organosilicate glass material with terminal methyl groups (30% porosity) deposited on top of Cu.

The film is uniform and has an amorphous structure; the pores are not visible.
